# Comorbidity of Epilepsy and Depression: Associated Pathophysiology and Management

**DOI:** 10.7759/cureus.21527

**Published:** 2022-01-23

**Authors:** Rasha Alhashimi, Sankeerth Thoota, Tejaswini Ashok, Vishnu Palyam, Ahmad T Azam, Oladipo Odeyinka, Ibrahim Sange

**Affiliations:** 1 Internal Medicine, University of Baghdad College of Medicine, Baghdad, IRQ; 2 Internal Medicine, Meenakshi Medical College Hospital and Research Institute, Kancheepuram, IND; 3 Internal Medicine, J.S.S. Medical College, Mysore, IND; 4 Internal Medicine, J.J.M. Medical College, Davangere, IND; 5 Internal Medicine, Allama Iqbal Medical College, Lahore, PAK; 6 Internal Medicine, University of Ibadan College of Medicine, Ibadan, NGA; 7 Research, K. J. Somaiya Medical College, Mumbai, IND

**Keywords:** management, pathophysiology, antidepressants, psychotherapy, depression, epilepsy

## Abstract

Epilepsy is a neurological disorder characterized by recurrent unprovoked seizures. Depression may arise as a result of other mental or physical problems or as a side effect of the drugs used to treat such illnesses, or it could be caused by epilepsy-related structural abnormalities. However, physicians are hesitant to prescribe antidepressants to patients with epilepsy due to concerns about decreasing seizure thresholds and the harmful drug interactions between antidepressants and antiepileptic medicines. As a result, the question about the optimal care of epileptic patients who suffer from depression remains unanswered. Despite the complicated link between epilepsy and depression, the co-administration of antidepressants and antiepileptic drugs (AEDs) is safe and beneficial when appropriately managed. A focused evaluation for depression (regardless of social, economic, or personal circumstances) might identify people who benefit from medical care and therapeutic assistance. Vagus nerve stimulation and psychological therapies such as cognitive-behavioral therapy, individual or group psychotherapy, patient support groups, family therapy, and counseling are nonpharmacological therapeutic alternatives. In terms of treatment strategy, it is critical to optimize seizure control and limit antiepileptic medications' adverse effects. Psychotherapy for depression in epilepsy is underused, even though it has been shown to be helpful in well-controlled studies. This review article has discussed some parts of the most common pathophysiologies of depression in patients with epilepsy, highlighted the efficacy of psychotherapy and antidepressant drugs, and explored the optimal care of patients with epilepsy who suffer from depression.

## Introduction and background

In 2005, epilepsy was described as a neurological condition characterized by an enduring predisposition for epileptic seizures. This term is commonly used in practice when having two unprovoked seizures separated by more than 24 hours [1]. As far back as the late Paleolithic period, seizures and epilepsy may have existed in some form [2]. Consistent with that period, people's understanding of seizures and their causes shifted over time from a magical to a scientific perspective [2]. The history of this sickness can be traced back to a tablet discovered in Mesopotamia, which describes an individual with a "neck turning, stiffness of hands and feet, wide-open eyes, and frothy secretion coming from the mouth without being conscious" [3]. The overall incidence rate of epilepsy was 61.4 per 100,000 person-years in a systematic review and meta-analysis of incidence studies (95% CI [50.7-74.4]) [4]. The overall lifetime prevalence of epilepsy was 7.60 per 1,000 population (95% CI [6.17-9.38]), with low- and middle-income countries (LMICs) having a higher prevalence (8.75 per 1,000; 95% CI [7.23-10.59]) than high-income countries (HICs) (5.18 per 1,000; 95% CI [3.75-7.15]) [5].According to some estimations, people of specific ethnicities are more likely to have the disease than others, and epilepsy is more common in people of Hispanic background than in non-Hispanics [6]. There were significant gender differences in the reporting of atonic seizures, which were more prevalent in males with generalized epilepsy (GE), and autonomic, visual, and psychological symptoms associated with non-acquired focal epilepsy (NAFE), which were more prevalent in females [6]. Epilepsy has a bimodal distribution related to age, with peaks occurring in both young and oldest [7]. The incidence increases with age, which can be linked to an increase in the number of age-related and aging-related epileptogenic disorders [7].

Epilepsy in adults is associated with many known risk factors, including head trauma, CNS infections, strokes (embolic and hemorrhagic), CNS malignant neoplasms, Alzheimer's disease, and other neurodegenerative disorders [8]. A seizure can be understood as a disruption of the brain's normal balance of excitation (E) and inhibition (I) [9,10]. This E/I imbalance can be caused by changes in various aspects of brain function, ranging from genes and subcellular signaling cascades to extensive neural circuits [10]. Factors affecting E/I balance might be either genetic or acquired. Genetic causes can manifest themselves in a variety of ways, ranging from faulty synaptic connections in cortical dysplasia to defective gamma-aminobutyric acid (GABA) receptor subunits. Similarly, acquired brain injuries can change the function of neural circuits [10]. Epilepsy can have severe consequences, including decreased lifespan, excessive body harm, neuropsychological and mental impairment, and social disabilities [11]. Seizures have been linked to brain injury, including neuronal death and physiological malfunction [11]. Mortality rates are four to seven times greater in patients with medically resistant seizures, and injury rates are high, ranging from one per 20 person-years to one per three person-years. Epilepsy impairs quality of life in many ways and is related to seizure control [11]. A thorough history and a general and neurological examination are required for an accurate diagnosis [12]. When interviewing witnesses to the seizure, more attention should be given to the possibilities of cyanosis, hypersalivation, tongue biting, and post-ictal disorientation, all of which are good signs of an epileptic genesis of the seizure [12]. The electroencephalogram (EEG) is used to diagnose epileptic seizures in children and adults [12]. If the EEG while awake is normal, a sleep EEG is recommended [12]. With more than 20 options of medications, up to 70% of newly diagnosed persons with epilepsy can be successfully managed [13]. Drugs that are used to treat epilepsy work by decreasing brain electrical activity, improving potassium channel function, suppressing excitation mediated by glutamate, or promoting inhibition mediated by GABA [13]. The efficacy of these drugs varies depending on the cause. Patients with no known etiology are more likely to be controlled, especially if their developmental history and neurological examination are normal [13].

Depression impairs the quality of life of epilepsy patients but is a curable illness. According to the latest research, the lifetime prevalence of depression in epilepsy is as high as 55%. Seizures can trigger depression via sleep deprivation, biological mechanisms, and the psychosocial consequences of epilepsy [14]. Numerous essential concerns remain unresolved as a result of the field's paucity of research [15]. Rather than a "one size fits all" approach, it is necessary to develop individualized treatment strategies depending on the type, frequency, and severity of seizures, as well as the degree and form of depressive symptoms [15]. On a more fundamental level, components of the biological and psychological interactions between the two illnesses must be identified to gain a deeper understanding of this complicated yet fascinating field of research [15]. This review article aims to: Highlight the pathophysiology of depression in patients with epilepsy, discuss various therapeutic modalities and their effectiveness in treating depression in patients with epilepsy, review types of treatment, including psychotherapy and antidepressants that can be used in these patients.

## Review

Depression and epilepsy: a shared pathophysiology

The relationship between depression and epilepsy is highly complicated and controversial. First, it was hypothesized that epilepsy caused depression. Still, new studies are now directed toward a bidirectional relationship between depression and epilepsy, the existence of shared pathogenic pathways that make the occurrence of one easier when the other is present [15]. A considerable proportion of patients with new-onset epilepsy were already depressed prior to their first seizure [16-18]. Another research study discovered that patients with epilepsy were 3.7 times more likely than the control group to have a history of depression prior to their initial seizure, correlating Hippocrates' clinical observations and implying a bidirectional association between epilepsy and depression [17]. In 96 epileptic patients from an outpatient setting, the relationship between depression and epilepsy was investigated by Indaco et al., as the study proposed that depression in epileptic patients is not a psychological reaction to a specific cognitive or physical disability but is instead associated with the type of epilepsy being experienced [19]. As in absence epilepsy, Moyanova and his colleagues studied the involvement of the melatoninergic system in the pathophysiology of spike and wave discharges (SWDs) and depression-like behavior in the absence type epileptic Wistar Albino Glaxo from Rijswijk (WAG/Rij) rat model. Their findings implied that a deficiency in the hippocampus melatoninergic system is one of the processes generating the depression-like phenotype in WAG/Rij mice and that activating melatonin receptors might be a useful method in treating depression associated with absence epilepsy [20].

**Figure 1:**
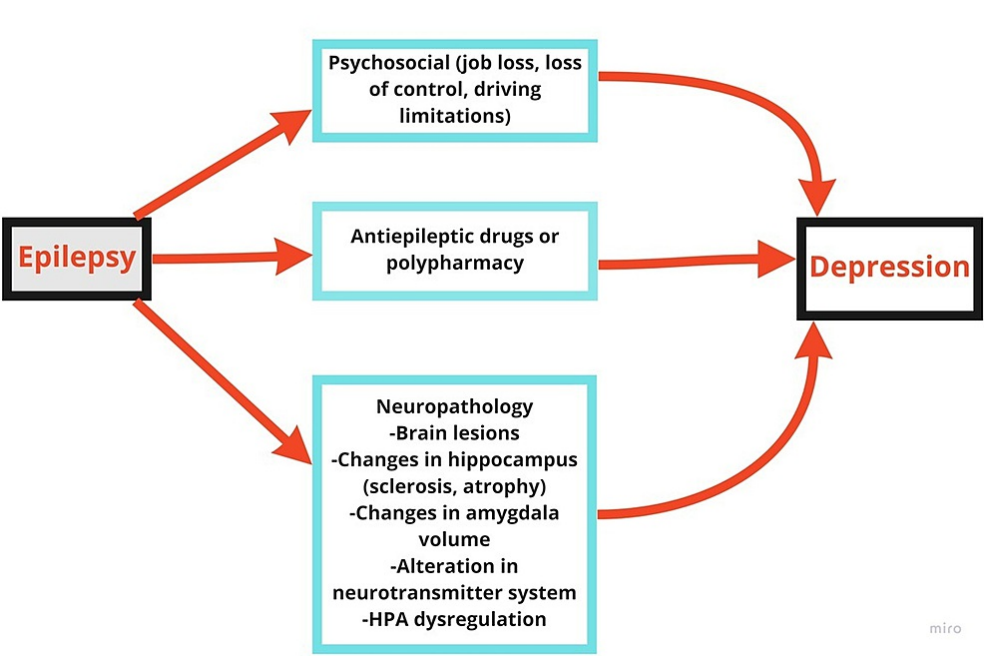
Summary of the pathophysiology of depression in patients with epilepsy HPA: Hypothalamic pituitary adrenal axis

Epilepsy of the temporal lobe (also known as temporal lobe epilepsy, or TLE) is a useful biological model for understanding the anatomical similarities between depression and epilepsy. TLE and clinical depression may be linked to an increase in hippocampus interleukin-1beta (IL-1beta) signaling as suggested by Mazarati et al. that increased IL-1B causes dysregulation in the hypothalamo-pituitary-adrenocortical axis (HPA) [21,22]. This dysregulation impaired raphe-hippocampal serotonergic transmission and led to depressive symptoms. Additionally, the purpose of this study by Mazarati was to determine if pharmacological inhibition of the hippocampus interleukin-1 receptor had antidepressant effects in an animal model of TLE and depression that occurred in Wistar rats following pilocarpine status epilepticus (SE) [21]. Bilateral intrahippocampal infusions of human recombinant Interleukin-1 receptor antagonist (IL-1ra) for two weeks alleviated all depression symptoms in naive rats without changing spontaneous seizure frequency or normal parameters. These findings link hippocampus IL-1beta in epilepsy-associated depression and support the use of IL-1beta antagonists in TLE treatment [21]. The temporal lobe epilepsy is divided into two types, mesial temporal sclerosis (MTS) and neocortical TLE. Quiske et al. conducted a study on 60 patients that revealed that MTS patients had significantly greater depression levels regardless of the lesion's lateralization. Depression was an excellent predictor of MTS but not the other way around. Thus, MTS might be considered a risk factor for developing mood problems in focal epilepsy (Figure 1) [23].

In a study done on 18 patients with TLE, Richardson et al. measured the resting metabolism of the amygdala and hippocampus. Their measurements revealed that both the right and left amygdala volumes are related to the degree of depression in individuals with TLE (Figure 1) [24]. Furthermore, the study suggested that depression in TLE patients would be associated with reduced hippocampus volumes and an increase in amygdala volumes [24]. Elst et al. also reported a correlation between amygdala volumes and depression [25]. On the other hand, animal studies on mice demonstrated that seizure-induced stimulation of the HPA axis increases seizure susceptibility and concomitant depression-like behaviors, implying that the HPA axis may represent a potential target for seizure management [26].

Management

When it comes to the treatment strategy, it is critical to maximize seizure control while minimizing unintended antiepileptic drug-related adverse effects as much as possible [27]. There is a significant underutilization of psychotherapy for depression in epilepsy (including online self-treatment programs) [27]. When commencing an antidepressant trial in a patient with epilepsy, three crucial factors must be considered: aggravation of seizure control, the possibility for interaction with antiepileptic drugs (AEDs), and antidepressant effectiveness in resolving depression symptoms [27].

Psychotherapy

Psychotherapy is a method of assisting people who are suffering from a wide range of mental diseases and emotional troubles. Psychotherapy can eliminate or manage troublesome symptoms, allowing a person to operate more effectively and experience more well-being [28]. Treatment of psychological illnesses and mental distress via the use of verbal and cognitive-behavioral approaches is referred to as psychotherapy in its general sense [28]. Cognitive-behavioral therapy (CBT) is defined as a type of psychotherapy that helps individuals recognize and alter harmful or ineffective thinking and behavior patterns, replacing them with more realistic beliefs and functional behavior patterns [29]. McLaughlin et al. looked at the effectiveness of a six-week group CBT treatment in community-dwelling persons with epilepsy over the age of 60. A total of 37 individuals were randomly allocated to one of two groups: CBT or control. Depression, dysthymia, psychosocial functioning, and seizure frequency were all assessed before and after the intervention [29]. Compared to the control group, the CBT group had a considerably lower seizure frequency (Cohen's d 0.63), but depression and psychosocial functioning scores did not differ between the groups (Table 1) [29]. The findings imply that additional research into the association between seizure frequency, psychological and psychosocial well-being in older persons is needed [29]. Chaytor et al. assessed another type of psychotherapy called Program to Encourage Active, Rewarding Lives (PEARLS), a home-based collaborative care solution for epilepsy patients that includes problem-solving, behavioral activation, and psychiatric counseling (Table 1) [30]. At baseline and at six, 12, and 18 months, patients were randomly randomized to PEARLS (N=40) or usual care (N=40) [30]. Over 18 months, patients allocated to PEARLS experienced decreased depression severity (P 0.05) (Hopkins Symptoms Checklist-20), decreased suicide thoughts (P 0.02), and improved emotional well-being (P 0.02), compared to those assigned to standard treatment. The PEARLS program dramatically reduced the symptoms of depression in persons with epilepsy, and that benefit persisted for 18 months from baseline and for more than a year after home visits are completed (Table 1) [30,31].

**Table 1:** Summary of included studies examining the efficacy of psychotherapy in patients with epilepsy CBT: Cognitive behavioral therapy, PHQ-9: Patient Health Questionnaire, PEARLS: A home-based collaborative care solution for epilepsy patients that includes problem-solving, behavioral activation, and psychiatric counseling, CBI; Cognitive-behavioral intervention, TAU: Treatment as usual, MDD: Major depressive disorder

References	Year	Design	Population	Method	Comments
McLaughlin et al. [29].	2011	Randomized trial	community-dwelling adults with epilepsy (60 years and above)	N=37 individuals were randomized to either a CBT or a control group.	The CBT group had a substantial reduction in seizure frequency compared to the control group. Depression and psychosocial functioning scores did not differ across groups.
Chaytor et al. [30].	2011	Randomized trial	people with epilepsy aged 18 or older who showed clinically significant depression on the PHQ-9 (PHQ-9)	PEARLS (N=40) or normal care (N=40)	baseline, 6, 12, and 18-month assessments Over 18 months, PEARLS patients had less depression, lesser suicide thoughts, and higher emotional well-being than standard care patients.
Martinović Z et al. [32].	2006	Randomized controlled trial	Adolescents with newly diagnosed epilepsy and increased risk for depression.	N=30, They were divided into two equal treatment groups: CBI (cognitive-behavioral intervention) and TAU (treatment as usual).	Subthreshold depressive disorder improved in the CBI group compared to the TAU group at follow-up.
Thompson et al. [33].	2015	Randomized, controlled crossover design	Adults with epilepsy and mild/moderate depressive symptoms from Georgia, Michigan, Texas, and Washington.	N=128, Participants were either allocated to Project UPLIFT or a TAU waitlist.	The intervention condition had a considerably lower incidence of MDD episodes (new or relapse) from baseline to interim evaluation (0.0 percent) than TAU (10.7 percent )
Gillham [35]	1990	Cross-over design	adult outpatients with poorly controlled epilepsy and significant psychological disorder	Two groups N = 19 and N = 21	The therapies were meant to teach coping skills. Weekly seizure frequency was tracked for 42 weeks, and depression was assessed before and after therapy. At 6-month follow-up, both groups exhibited a substantial reduction in seizure frequency. Psychological symptoms improved significantly on self-rating scales.

In adolescents, cognitive-behavioral intervention (CBI) was assessed by Martinović et al., 30 patients (mean age 17.4, 60% females) were randomly assigned to one of two equal treatment groups: cognitive-behavioral intervention (CBI) or treatment as usual (TAU) [32]. Compared to the TAU group, the CBI group showed a significant improvement in subthreshold depressive disorder at follow-up (P 0.05) (Table 1) [32]. In another study, Thompson et al. did a weekly Project UPLIFT (Using Practice and Learning to Increase Favorable Thoughts), an intervention based on mindfulness-based cognitive therapy was administered in a group setting over the Internet or telephone for eight sessions [33]. Participants were randomly allocated to either Project UPLIFT or the TAU waitlist. They were evaluated at baseline and after intervening in the intervention group (for approximately 10 weeks) and in the TAU group (for approximately 20 weeks) using a randomized, controlled crossover design [33]. Valid self-report measures of depression (modified version of the Beck Depression Inventory, 6-item Neurological Disorders Depression Inventory for Epilepsy, patient health questionnaire-9) and knowledge/skills, and life satisfaction were used in the evaluations of the participants [33]. The study showed that the depressive symptoms dropped considerably more in the intervention condition than in the TAU condition; there was no difference between the web and telephone conditions. Changes in knowledge/skills mediated the impact, which lasted for the whole 10-week follow-up period. Knowledge/skills and life satisfaction improved much more in the intervention arm than in the TAU arm (Table 1) [33].

Tan et al. conducted a clinical trial on 27 outpatients divided randomly into one of three groups: Cognitive-Behavioral Therapy, Supportive Counseling (attention-placebo control), or Waiting list (no treatment control). The following outcome measures were used: global ratings of psychological adjustment by the patient, neurologist, and therapist, patient target complaints and weekly seizure frequency, patient and neurologist ratings of seizure control, the Minnesota Multiphasic Personality Inventory, the Washington Psychosocial Seizure Inventory, and the Beck Depression Inventory [34]. There were no significant differences in these measures between the three groups except for therapists' global ratings of psychological adjustment, which showed significant improvements in both the Cognitive-Behavior Therapy and Supportive Counseling groups but not in the Waiting List control group following therapy [34].

psychotherapy showed improvement in epilepsy control and decreased the frequency of the attacks, as displayed by Gillham et al. study [35]. Two groups of adult outpatients (n = 19 and n = 21) with poorly managed epilepsy and severe psychological disturbance received two psychological therapies in a balanced cross-over design. After establishing a stable baseline, a third group (n = 19) of patients with poorly controlled epilepsy but no severe psychological disturbance underwent one form of psychological treatment. The therapies were educational in nature and aimed to boost coping abilities [35]. For 42 weeks, seizure frequency was evaluated weekly, and self-report measures of anxiety and depression were gathered before and after therapy. All three groups experienced a substantial decline in seizure frequency. The two groups with psychological symptoms both improved significantly on self-rating scales (Table 1) [35]. In students, a controlled study showed effective skill training is required for the therapy of depression in an epilepsy population. With 13 chronically depressed epileptic students (10 females and three males, with a mean age of 33.1 years), cognitive-behavioral approaches were used in a structured learning manner [36]. Significant reduction in dysphoria/depression was observed among the students in the treatment group than among the students in the control group, as judged by the Depression Adjective Checklist and the Generalized Contentment Scale. The Community Adjustment Questionnaire revealed significant decreases in anger and anxiety/stress, as well as increases in social activities [36].

The pharmacological management 

Depression is the most prevalent mental comorbidity in persons with epilepsy, impacting approximately one-third of patients and negatively influencing the quality of life [37]. There is a worry that individuals may not be receiving adequate therapy for depression due to confusion about which antidepressant or class of antidepressants works best and the perceived danger of aggravating seizures.

AEDs and vagus nerve stimulation (VNS) therapy, should be used initially in patients with depression and epilepsy [38]. There is evidence that several anticonvulsants (valproate, carbamazepine, lamotrigine, and gabapentin) may help improve mood in epilepsy patients [38]. However other anticonvulsants such as levetiracetam may cause behavioral changes (positive or negative) [39]. When antidepressants are needed to treat depression in epilepsy patients, selective serotonin reuptake inhibitors (SSRIs) and multi receptor antidepressants (MRAs) are considered first-line therapy [38]. Antidepressant medicines' complicated neurotransmitter activities make it hard to draw straightforward generalizations regarding their proconvulsant effects [40]. Recent experimental research on AEDs used to treat depression suggested that changes in serotonin and norepinephrine levels are unlikely to be associated with an increased risk of seizures. Indeed, certain investigations indicated that doxepin might exhibit anticonvulsant activity on occasions [40]. Ajinkya et al. found that screen positive depressive symptoms (SPDS) affect a quarter of adults with epilepsy (AWE) in the United States, although only half receive therapy [41]. This showed that more needs to be done to screen for and treat depression in AWE [41,42].

Sertraline (SRT) was used in a study to treat depressive (N = 97) or obsessive-compulsive (N = 3) disorders [43]. Kanner et al. compared the monthly seizure frequency observed while on SRT to that reported in the three and 12 months prior to SRT initiation. They concluded that SRT might be used safely in the great majority of epileptic patients (Table 2) [43]. The research by Specchio et al. included depressed epileptic patients who were on AEDs. Patients with a low frequency of seizures were treated for four months with citalopram (20 mg/d). A change in seizure frequency from baseline was used to assess citalopram's safety, while depressive symptoms were used to assess the drug's efficacy [44]. Clinical evaluations were conducted at baseline and after two and four months of citalopram medication, respectively. Overall, the 39 individuals who completed the research had a decrease in seizure frequency. Plasma AED concentrations remained stable during medication management, and depression symptoms significantly improved (Table 2) [44]. Kühn et al. conducted a study on three antidepressants drugs. Seventy-five patients were given conventional care with citalopram, mirtazapine, or reboxetine at the indicated dosage. At the time of admission, the Hamilton Rating Scale for Depression was used, as well as after four and 20-30 weeks. Anticonvulsant plasma levels were measured at admission and discharge (Table 2) [45]. Seizures were recorded. All antidepressant groups found the therapy to be effective. There were no reports of major adverse effects or medication interactions. Seizures did not become more frequent or more severe. Mirtazapine had a considerably greater dropout rate than reboxetine or citalopram at the endpoint (one probable explanation is that mirtazapine's side effects, such as sleepiness and weight gain, are particularly unpleasant for the patients since some anticonvulsants have similar effects). At week four, reboxetine appeared to be more effective than citalopram but not mirtazapine (Table 2) [45].

**Table 2:** Summary of included studies examining the efficacy of antidepressants in patients with epilepsy OCD: Obsessive-compulsive disorder, MDD: Major depressive disorder, TLE: Temporal lobe epilepsy, SSRI: Selective serotonin reuptake inhibitors, SNRI: Serotonin and norepinephrine reuptake inhibitors

References	Year	Design	Population	Method	Comments
Kanner et al. [43]	2000	Prospective controlled trial	Patients with partial or generalized epilepsy and depression or OCD	N=95 partial epilepsy, N=5 generalized epilepsy, N=97 depressive disorder , N=3 OCD	Sertraline is safe to use in epilepsy patients
Specchio et al. [44]	2004	Open, multicentered, uncontrolled clinical trial	Depressed epileptic patients on antiepileptic drugs	N=45, Six patients dropped out	Four months of citalopram (20 mg/d) therapy improved depression symptoms and reduced seizure frequency.
Kühn et al. [45]	2003	Clinical trial	Inpatients with MDD and TLE	N=75 received standard treatment with citalopram, mirtazapine, or reboxetine, respectively	At Week 4, reboxetine was shown to be more effective than citalopram but not mirtazapine.
Ribot et al. [47]	2017	Retrospective observational study	Patients with epilepsy	N=100 were started on an SSRI or SNRI	SSRIs and SNRIs did not appear to increase seizure frequency. Also, regardless of seizure frequency, these medications appeared to treat mental symptoms effectively.
Thomé-Souza et al. [48]	2007	Clinical trial	children and adolescents with epilepsy and depressive disorders	N=36 were started on Sertraline or fluoxetine	SSRIs were an effective treatment choice, with low side effects and good seizure control in children and adolescents.

The strongest evidence for effectiveness and safety now supports using citalopram, sertraline, or mirtazapine as first-line pharmacotherapy, but bupropion should be avoided. Always remember to begin slowly and to use the lowest effective dosage. Vagus nerve stimulation appeals to persons with refractory partial epilepsy and refractory depression since it may be suitable for both disorders. However, the efficacy of vagus nerve stimulation in relieving mood in patients with epilepsy remains unknown [46]. Ribot et al. agreed with all the studies above and found that SSRI and serotonin-norepinephrine reuptake inhibitors (SNRIs) are effective in treating depression and did not increase the seizure frequency (Table 2) [47]. Thomé-Souza et al. found that SSRIs are a practical treatment choice for children and adolescents with epilepsy and depressive disorders, given their ability to alleviate depressive symptoms, their low risk of side effects, and their ability to control seizures at an acceptable level (Table 2) [48]. Pharmacotherapy, coupled with non-pharmacological therapy, can be an effective tool to treat depression in patients with epilepsy.

Limitations 

This study does not take into account the genetic susceptibility to depression, as well as environmental variables that may enhance the susceptibility to depression in people with epilepsy. Furthermore, when evaluating the efficacy of treatment, the study did not consider the severity of the depression presentation at the time of the trial. Lastly, the sample sizes in all of the studies were relatively small.

## Conclusions

Patients with epilepsy frequently suffer from depression, which is a prevalent psychiatric disorder in this population. When it comes to depression and epilepsy, it appears that the relationship is bidirectional; having depression increases the risk of developing epilepsy, and having epilepsy appears to increase the risk of developing depression. In light of these considerations, effective and safer treatment of depression associated with epilepsy is of crucial clinical importance. A significant number of studies have revealed that psychotherapy effectively treats depression in patients with epilepsy. It might be challenging to know where to start when it comes to pharmacological therapy of individuals with epilepsy and mental comorbidities, although modern antidepressants (SSRIs, SNRIs) are regarded to be safe and effective for usage in epilepsy. Finally, we recommend that more important studies be performed on the relationship between epilepsy and depression to establish a more coordinated and directed approach for detecting and treating depression.
